# Instrument care: everyone's responsibility

**Published:** 2011-12

**Authors:** Renée du Toit, Konio Szetu, Wanta Aluta, Alumita Ravono

**Affiliations:** Professional Development Director, The Fred Hollows Foundation New Zealand. Email: rdutoit@hollows.org.nz; Senior Nurse Manager, The Pacific Eye Institute, The Fred Hollows Foundation New Zealand. Email: kszetu@hollows.org.nz; National Eye Care and Training Co-ordinator, Solomon Islands Ministry of Health and Medical Services, Solomon Islands. Email: alutawanta@yahoo.com.au; Nursing Clinical Supervisor, The Pacific Eye Institute, The Fred Hollows Foundation New Zealand. Email: alumitaravono@yahoo.com

**Figure:**
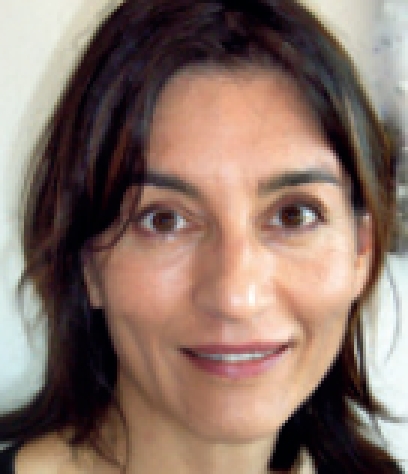
Renée du Toit

**Figure:**
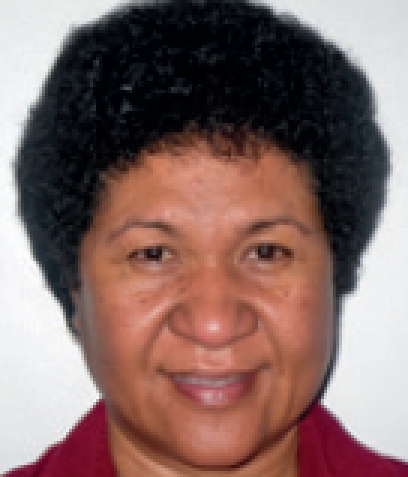
Konio Szetu

**Figure:**
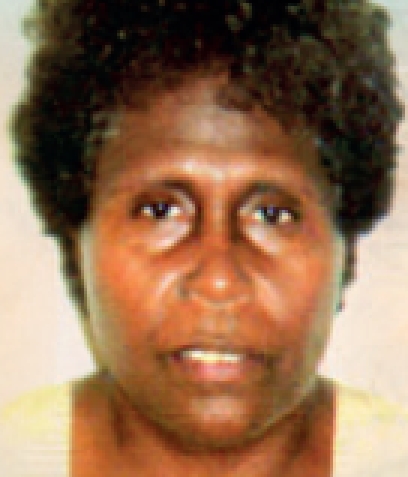
Wanta Aluta

**Figure:**
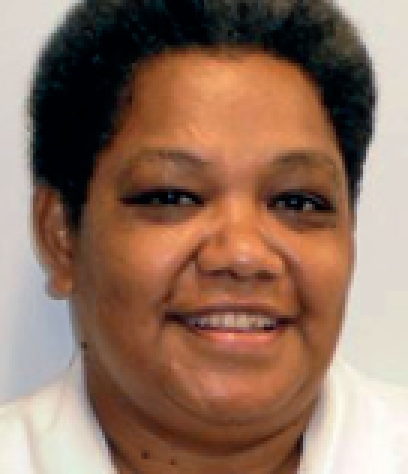
Alumita Ravono

Some eye units have technicians who are responsible for taking care of instruments. It is certainly important to have a specific person assigned to do tasks such as monthly checking and maintenance, even on a part-time basis.

However, everyone working in an ophthalmic operating theatre must be competent in the care, handling, storage, and maintenance of instruments. This will help to improve surgical outcomes, maintain an economic and affordable service for patients, and provide a safe environment for the wellbeing of patients and staff.

Including instrument care in theatre courses and in-service training is one way of ensuring staff competence. Table 1 opposite provides some guidance about the different skills each team member must be able to master.

To support in-service training, we suggest that you provide staff with lists of duties, protocols, and guidelines for instruments. This ensures that everyone will adhere to the same standards when they demonstrate instrument handling, care, and maintenance to learners. These documents can also serve as a reference for learners.

Support learners by encouraging staff to provide close supervision and give constructive (positive, supportive, and informative) feedback.

## A supportive environment

Training by itself is not enough. Staff require an environment that supports them to take good care of instruments and provide high-quality care in the operating theatre. The different components of the health system (human resources, finance, equipment and supplies, leadership and governance, and health information systems) provide a useful framework

### Human resources

- Plan for sufficient trained personnel to work in the operating theatre; this ensures that the standards of instrument care are maintained.
- Include a section on instrument care in the job descripitions of staff. Job descriptions document the roles and responsibilities of each person in the operating team and can be used as a basis for evaluating staff performance. You can then evaluate (and reward) staff competence in instrument care.
  **Table 1:** Roles of the eye care team members in looking after instruments

**Instrument cleaning nurse/technician**	**Circulating nurse (or equivalent)**	**Surgical assistant/scrub nurse (or equivalent)**	**Surgeon**
**To ensure safety**			
careful passing and placement of instruments, e.g., onto cleaning dishes and instrument trays	careful passing to the instrument cleaning nurse or technician	careful handling to and from the surgeon	careful handing to and from the surgical assistant/scrub nurse
**To enhance efficiency and facilitate quality outcomes**			
place instruments in order of use	collect used instruments and take these to the cleaning area	collect the used instruments immediately after surgery, take to the cleaning area	use instruments for their intended purpose
**To maintain sterility**			
follow recommended temperatures and times for autoclaving and/or soaking	check sterility indicators take care when opening the autoclave, placing instruments on sterile trolley ensure that sterile water is ready for rinsing instruments that have been soaked	careful passing to surgeon, using aseptic non-touch technique ensure that instruments that have been sterilised in a sterilisation solution are thoroughly rinsed before use	handle and use instruments properly for their intended purpose
**To prevent loss and preserve functionality of instruments**			
account for all instruments check instruments before putting them away: tips, sharpness, rust, functional springs, hinges, and damage, ideally with magnification dry instruments before storing on clean, closed shelves in a secure but ventilated environment	keep a list of damaged instruments and inform the nurse/technician, so that these instruments can be repaired or replaced as soon as possible do not keep defective instruments in surgical sets ensure all instruments are accounted for	ensure all instruments are accounted for, especially when removing drapes from the trolley ensure that instrument sets are correct, with the known number and type of instrument in place	use instruments for their intended purpose report defective instruments
**To maintain instruments and prevent damage**			
clean instruments individually use correct solutions in appropriate concentrations and containers for soaking and cleaning place instruments carefully, without piling, in cleaning and rinsing dishes, the autoclave, and/or the sonic cleaner protect the tips of sharp instruments when packing or storing them	careful handling and proper passing, especially when instruments are not packed in an instrument tray	handle instruments properly during cleaning and surgery wipe instruments during surgery if required carefully place instruments next to each other on the trolley group different types of instruments together	handle instruments as per protocol avoid throwing instruments down, hand them to the scrub nurse instead
- Provide continuing professional development in instrument care. For example, ask staff to teach skills they know well to small groups of co-workers, or ask staff to reflect on their instrument care and come up with ways of improving what they do. You can also encourage staff to read and discuss articles on equipment care such as those in this journal (see page 44 of this issue, as well as previous issues).

### Finance

- Purchase the best quality instruments that your eye centre can afford as these are likely to last longer and may contribute to better quality outcomes
- Purchase sufficient instruments for the number of patients seen in your unit
- Purchase appropriate instruments for different procedures
- Allocate funds for replacement of defective instruments.

### Equipment and supplies

- Develop and implement protocols for instrument maintenance and care
- Schedule monthly maintenance procedures, including a check of the functionality of the instruments.

### Leadership and governance

- Ensure that your eye unit complies with standards of occupational health and safety, theatre design and layout, materials, and infrastructure.
- Develop and implement checklists from best practice and evidence-based standards, guidelines, or protocols to cover the following areas: equipment, instruments, infection control, documentation, administration, stock management, productivity, and the roles and responsibilities of staff.

### Health information systems

- Report defective instruments to the person responsible on a regular basis, ideally at the end of the day or of the operating list.

### Service delivery

- The quality of care during outreach visits should be maintained at similar standards to a permanent facility. This means taking the same level of care with instruments and adhering to the same high standards of disinfection. Ensure instruments are packed carefully for transport.

